# Update on the Prevalence of the PCV2 Major Genotypes PCV2a, PCV2b, and PCV2d in German Fattening Farms in 2024

**DOI:** 10.3390/vetsci12080717

**Published:** 2025-07-30

**Authors:** Matthias Eddicks, Sarah Ladurner Avilés, Stefanie Frauscher, Roman Krejici, Sven Reese, Robert Fux, Mathias Ritzmann

**Affiliations:** 1 Clinic for Swine at the Centre for Clinical Veterinary Medicine, Ludwig-Maximilians-Universität München, 85764 Oberschleissheim, Germany; 2 Ceva Sante Animale, 33501 Libourne, France; 3 Institute for Anatomy, Histology and Embryology, Department of Veterinary Sciences, Ludwig-Maximilians-Universität München, 80539 München, Germany; 4 Division of Virology, Institute for Infectious Diseases and Zoonoses, Department of Veterinary Sciences, Ludwig-Maximilians-Universität München, 85764 Oberschleissheim, Germany

**Keywords:** prevalence, porcine circovirus, genotyping, vaccination

## Abstract

The occurrence of PCV2 genotypes in domestic pig production is a dynamic process that undergoes continuous change. Beginning with PCV2a as the first recognized genotype, PCV2b, and subsequently PCV2d, has become the most prevalent one over time. The present study provides an update on the prevalence of the three major PCV2 genotypes in Germany in 2024. A total of 87 fattening farms were randomly selected, proportionally based on farm density within the respective federal states. On each farm, oral fluid samples (OFs) were collected from approximately 100 pigs aged 18 (±1) weeks. Oral fluids (OFs) were pooled and screened for PCV2 DNA by qPCR. Positive samples were subsequently examined by genotype specific qPCR. In total, 31.0% (27/87) of all farms were identified as PCV2-positive. PCV2a was detected in 8.0% (7/87) of farms, while 3.4% (3/87) tested positive for both PCV2a and PCV2d. Overall, 11.5% (10/87) of all farms were PCV2d-positive. No significant effect of vaccination status of the pigs on the viral load or frequency of detection of PCV2 DNA was detected.

## Simple Summary

Porcine circovirus-2 (PCV2) still remains a challenge for the health of pigs. Within the last years, nine PCV2 genotypes (a–i), with different regional patterns of occurrence, were described. However, PCV2d was reported as the most prevalent one in the domestic pig population globally. Within the present study, we collected oral fluids (OFs) from 87 randomly selected fattening farms in Germany. The OFs were screened for PCV2 and, in case of a positive result, subsequently genotyped by qPCR to determine the PCV2 genotype. As expected, PCV2d was the most often detected genotype at farm level, whereas PCV2a alone or in combination with PCV2d was unexpectedly detected often. PCV2b was not present or remained under the limit of detection in the sample collection. The study results show the dynamic in the occurrence of PCV2 genotypes at a regional level and point out, that the ongoing genotype shift is rather from PCV2b to PCV2d whereas PCV2a still circulates on a relevant level in German domestic farms fattening pigs.

## 1. Introduction

The porcine circovirus 2 (PCV2) is associated with numerous diseases and disease syndromes called porcine circovirus diseases (PCVD) in pigs of all ages. Among these, the post-weaning multisystemic wasting syndrome (PMWS) is considered the most economically significant, as it leads to increased mortality and reduced performance in affected populations. Moreover, reproductive disorders (PCV2-reproductive disease) or the porcine dermatitis and nephropathy syndrome (PDNS) can be recognized in pigs affected by relevant PCV2 infections [1,2]. However, subclinical PCV2 infection (PCV2-SI) seems to be the most often occurring form of PCV2 infection in pigs [1]. Although vaccination against PCV2 is regarded as an effective measure to control PCVD [3,4,5,6,7], the virus still remains a major threat to pig health, especially in cases where the vaccination does not reach full efficacy [8,9,10,11]. PCV2 can be assigned to nine genotypes (PCV2a-i) [12,13]. Over the past two decades, two major genotype shifts have been observed. The first around 2005/2006, when PCV2b displaced PCV2a as the predominant genotype in clinical samples [14,15,16], and the second from 2010 onward, when PCV2d emerged as the most prevalent genotype [8,9,17,18]. Besides the examination of serum for PCV2 DNA by real time PCR, oral fluids (OFs) have become a popular diagnostic specimen for PCV2 DNA detection [19,20] as well as for downstream applications such as sequencing or genotyping by PCR. In a study by Nielsen et al. [19], OFs even demonstrated a higher sensitivity for PCV2 DNA detection compared to pen-wise pooled serum samples. The present study aimed to provide an update on the currently circulating PCV2 genotypes in the German fattening pig population in 2024.

## 2. Material and Methods

### 2.1. Study Farms and Sampling

To enable the random selection of study farms, bundlers, and producer cooperatives from all federal states of Germany were asked to deliver anonymized lists of fattening farms as potential study participants. To ensure blinding, neither the objective of the study nor specific details were disclosed to these partners or the farmers. A total of 401 fattening farms were available for random selection. The final selection of study farms, stratified by federal state, was carried out using an online randomization tool (randomizer.org). Among the study farms, 93.1% (81/87) housed PCV2-vaccinated pigs (V), 4.6% (4/87) housed non-vaccinated pigs (N), and for 2.3% (2/87) of the farms, the vaccination status was unknown (U).

According to the principles of binomial distribution, in a pen of 20 animals with at least 10% of animals shedding PCV2, a PCV2-positive rope can be identified with a probability of 87.8%. If five pens are randomly selected and tested per farm, a PCV2-positive herd will be identified as such with a probability of 99.9%. This sampling strategy allows for the detection of PCV2-positive farms with a 95% confidence level. To estimate the prevalence with a 95% confidence interval and a maximum margin of error of ±10% points, a total of 87 farms were sampled, meeting the required minimum level of precision for prevalence estimation.

Oral fluid samples were collected using the Oral Fluid Sample Collection Accessory Kit (IDEXX, Scorpius 60 Building F, Hoofddorp, The Netherlands). Samples were collected from pigs with an age of approximately 18 weeks (+/− 1 week). The collection procedure followed previously published protocols. In brief: the aim was to include approximately 100 pigs per farm with 20 pigs sampled per rope. Consequently, the number of collected ropes depended on the number of pigs per pen. If more than 20 animals were housed in one pen, two or more ropes were placed accordingly. The cotton ropes remained in the pens for 25 min.

### 2.2. Laboratory Diagnostics

Screening for PCV2 DNA and subsequent genotyping was performed using a qPCR [21] or genotype specific qPCR [22] as described elsewhere. For the initial qPCR screening, a cycle quantification (Cq) value of below 40 was defined as positive. All samples testing positive in the qPCR screening were subsequently analyzed using the genotype-specific qPCR assays. 

### 2.3. Statistical Analysis

Gained data were documented in Excel as a metric scale (Cq-values) or nominal data (PCV2 PCR: positive/negative, PCV2 vaccination of the pigs: yes/no). Nominal data were tested for associations using the Chi^2^ Test and odds ratio. Metric data (Cq-values) were tested for normal distribution using the Kolmogorov–Smirnov Test and tested for associations using ANOVA with Bonferroni corrections for multiple comparisons regarding the PCV2 genotype results.

## 3. Results

In total, 31.0% (27/87) of all farms were identified as PCV2-positive. PCV2 was detected in 30.9% (25/81) of the V-farms, 50.0% (2/4) of the N-farms, and 0.0% (0/2) of the U-farms.

**Figure 1:**
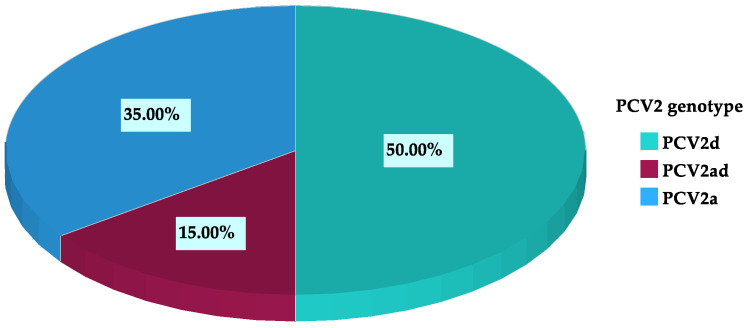
PCV2 genotype distribution at farm level within all genotyped farms (*n* = 20).

A PCV2 genotype could be assigned for 20 out of the 87 farms. Among all farms, 11.5% (10/87) were PCV2d-positive, 8.0% (7/87) were PCV2a-positive, and 3.4% (3/87) were positive for both PCV2a and PCV2d. Within the farms that were genotyped, 50.0% (10/20) were PCV2d-positive, 35.0 % (7/20) were PCV2a-positive, and 15.0% (3/20) were positive for both genotypes (Figure 1).

In relation to vaccination status, among the V-farms with genotype data (*n* = 18), 33.3% (6/18) were PCV2a-positive, 50.0% (9/18) PCV2d-positive, and 16.7% (3/18) positive for both genotypes (PCV2a and PCV2d). Among the N-farms with genotype data (*n* = 2), 50.0% (1/2) were PCV2a-positive and 50.0% (1/2) PCV2d-positive. No significant association between vaccination status and frequency of PCV2-positive farms were observed. PCV2 detection by federal state and assigned PCV2 genotypes are available in Table 1.

### Metric Analysis of Oral Fluids Pools

**Figure 2:**
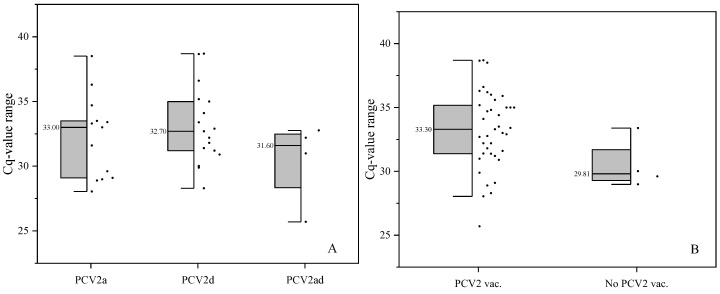
Half boxes with median of Cq values of OFs assignable to different PCV2 genotypes (**A**) or vaccination status (**B**) of the pigs (only PCV2-positive pools included).

A total of 157 OF pools were included in the analysis. Of these, 43 pools (27.4%) were PCV2 DNA-positive. No significant difference was observed in the Cq values between the different PCV2 genotypes (Figure 2A; *p* = 0.173). However, farms from group N had tendentially lower Cq values via ANOVA compared with farms from group V (Figure 2B; *p* = 0.079; only PCR-positive pool included).

## 4. Discussion

The present study presents randomly obtained data on the prevalence of the three major PCV2 genotypes PCV2a, PCV2b and PCV2d in the OFs of fattening pigs collected in Germany in 2024. The overall PCV2 prevalence, as determined by OF sampling, was lower than expected, given that PCV2 is considered to be ubiquitous in pig production [23]. As the overall prevalence of PCV2-positive farms may be higher when all stages of production are considered, our findings are limited to the specific age group examined in this study. Nevertheless, the last third of the fattening period appears to be suitable to sample fattening pigs, particularly in settings with high vaccination coverage [3].

Moreover, seroprevalence was not examined in this study, as the high vaccination coverage among the farms would likely limit the value of such an analysis. The usefulness of OFs for PCV2 screening purposes was already demonstrated by Nielsen et al. [19], who showed that OFs might even have greater sensitivity compared with pooled serum samples. Although qPCR for genotyping PCV2 exhibits a higher sensitivity for that purpose compared with sequencing in cases with low viral loads [22], we were able to assign PCV2 genotype(s) to 20/27 PCV2-positive farms only. As can be referred from the results, the low viral loads in the OFs of some farms were probably below the limits of capacity of the qPCR used.

In the present study, PCV2d was identified as the most prevalent genotype in German fattening farms, whereas PCV2b could not be detected in the sampled study population. These findings are consistent with those of Dei Giudici et al. [24], who reported only a few PCV2b sequences in a large-scale study on PCV2 genotypes conducted in Sardinia. One possible explanation for the high PCV2d prevalence might be differences in monocyte uptake of the different PCV2 strains in association with the amino acid characteristics on the surface of their capsid, which might lead to a selection advantage for PCV2d as shown by others [25].

However, we also observed an unexpectedly high PCV2a prevalence in our sample collection. These findings may be attributed to the ability of qPCR to detect multiple PCV2 genotypes within one sample, in contrast to classical sequencing methods [22]. Moreover, the random sampling strategy might also contribute to different prevalence data compared with clinical samples which are affected by sample bias. Overall, our findings support the observation of a PCV2 genotype shift from PCV2b to PCV2d, while also indicating a continued circulation of PCV2a. Neither the vaccination status nor the PCV2 genotype present in the OFs were significantly associated with the Cq-value obtained by qPCR. Although PCV2 vaccination is known to reduce the viral load in blood or prevent viremia [3,6], potentially even below the detection limit [4], the random farm selection and the low number of farms with unvaccinated pigs likely limited the ability to draw conclusions regarding vaccine effects in this study. According to Nielsen et al. [19], the viral load in OFs can be one log higher than in serum samples. Therefore, the observed Cq-values in the OFs indicate an overall low viral burden in the study farms, indicating subclinical infection.

Differences in the PCV2 viral load between genotypes may be influenced by co-infections. In co-infection models involving PCV2 and PRRSV, PCV2d exhibited a higher viral load compared to PCV2a or PCV2b [26]. Comparable results were achieved by Oh et al. [27] in a co-infection model with *Mycoplasma (M.) hyopneumoniae*. These results indicate that PCV2d might have a higher clinical relevance in co-infection scenarios. However, no information on the *M. hyopneumoniae* or PRRSV status of the farms was available in the present study, and no PCV2 genotype-specific effect on the viral load in OFs was observed.

## 5. Conclusions

Within the randomly conducted OF sample collection from 2024, PCV2d was identified as the most prevalent genotype, followed by PCV2a. PCV2b was not detected in any of the samples. These findings suggest that PCV2b might have been displaced by PCV2d. However, the continued detection of PCV2a indicates that this genotype is still circulating within German commercial pig farms.

## Figures and Tables

**Table 1:** PCV2 prevalence in different federal states of Germany sorted by percent positives and PCV2 genotypes assigned to farms.

Federal State	Number of Study Farms (*n* = 87)	PCV2-Positive Farms	PCV2 Genotype Assigned to Farms if Typeable
Schleswig-Holstein	3	66.7% (2/3)	PCV2d (2/2)
North Rhine-Westphalia	27	44.4% (12/27)	PCV2a (4/7) PCV2a + PCV2d (1/7) PCV2d (2/7)
Hesse	3	33.3% (1/3)	No typeable farms
Lower Saxony	22	31.8% (7/22)	PCV2a (2/7) PCV2a + PCV2d (2/7) PCV2d (3/7)
Bavaria	18	22.2% (4/18)	PCV2a (1/3) PCV2d (2/3)
Baden-Wuerttemberg	8	12.5% (1/7)	PCV2d (1/1)
Brandenburg	1	0% (0/1)	-
Rhineland-Palatinate	1	0% (0/1)	-
Saxony	1	0% (0/1)	-
Saxony-Anhalt	1	0% (0/1)	-
Mecklenburg-Western Pomerania	1	0% (0/1)	-
Thuringia	1	0% (0/1)	

## Data Availability

Data can be obtained from the corresponding author after reasonable request.

## References

[1] Segales J. (2012). Porcine circovirus type 2 (PCV2) infections: Clinical signs, pathology and laboratory diagnosis. Virus Res..

[2] Segales J., Sibila M. (2022). Revisiting Porcine Circovirus Disease Diagnostic Criteria in the Current Porcine Circovirus 2 Epidemiological Context. Vet. Sci..

[3] Kixmoeller M., Ritzmann M., Eddicks M., Saalmueller A., Elbers K., Fachinger V. (2008). Reduction of PMWS-associated clinical signs and co-infections by vaccination against PCV2. Vaccine.

[4] Feng H., Blanco G., Segales J., Sibila M. (2014). Can Porcine circovirus type 2 (PCV2) infection be eradicated by mass vaccination?. Vet. Microbiol..

[5] Fachinger V., Bischoff R., Jedidia S.B., Saalmueller A., Elbers K. (2008). The effect of vaccination against porcine circovirus type 2 in pigs suffering from porcine respiratory disease complex. Vaccine.

[6] Fort M., Sibila M., Allepuz A., Mateu E., Roerink F., Segales J. (2008). Porcine circovirus type 2 (PCV2) vaccination of conventional pigs prevents viremia against PCV2 isolates of different genotypes and geographic origins. Vaccine.

[7] Fort M., Sibila M., Perez-Martin E., Nofrarias M., Mateu E., Segales J. (2009). One dose of a porcine circovirus 2 (PCV2) sub-unit vaccine administered to 3-week-old conventional piglets elicits cell-mediated immunity and significantly reduces PCV2 viremia in an experimental model. Vaccine.

[8] Eddicks M., Fux R., Szikora F., Eddicks L., Majzoub-Altweck M., Hermanns W., Sutter G., Palzer A., Banholzer E., Ritzmann M. (2015). Detection of a new cluster of porcine circovirus type 2b strains in domestic pigs in Germany. Vet. Microbiol..

[9] Opriessnig T., Xiao C.T., Gerber P.F., Halbur P.G. (2013). Emergence of a novel mutant PCV2b variant associated with clinical PCVAD in two vaccinated pig farms in the U.S. concurrently infected with PPV2. Vet. Microbiol..

[10] Seo H.W., Park C., Kang I., Choi K., Jeong J., Park S.J., Chae C. (2014). Genetic and antigenic characterization of a newly emerging porcine circovirus type 2b mutant first isolated in cases of vaccine failure in Korea. Arch. Virol..

[11] Opriessnig T., O’Neill K., Gerber P.F., de Castro A.M., Gimenez-Lirola L.G., Beach N.M., Zhou L., Meng X.J., Wang C., Halbur P.G. (2013). A PCV2 vaccine based on genotype 2b is more effective than a 2a-based vaccine to protect against PCV2b or combined PCV2a/2b viremia in pigs with concurrent PCV2, PRRSV and PPV infection. Vaccine.

[12] Wang Y., Noll L., Lu N., Porter E., Stoy C., Zheng W., Liu X., Peddireddi L., Niederwerder M., Bai J. (2020). Genetic diversity and prevalence of porcine circovirus type 3 (PCV3) and type 2 (PCV2) in the Midwest of the USA during 2016–2018. Transbound. Emerg. Dis..

[13] Franzo G., Tucciarone C.M., Legnardi M., Drigo M., Segales J. (2024). An updated phylogeography and population dynamics of porcine circovirus 2 genotypes: Are they reaching an equilibrium?. Front. Microbiol..

[14] Gagnon C.A., Tremblay D., Tijssen P., Venne M.H., Houde A., Elahi S.M. (2007). The emergence of porcine circovirus 2b genotype (PCV-2b) in swine in Canada. Can. Vet. J..

[15] Cheung A.K., Lager K.M., Kohutyuk O.I., Vincent A.L., Henry S.C., Baker R.B., Rowland R.R., Dunham A.G. (2007). Detection of two porcine circovirus type 2 genotypic groups in United States swine herds. Arch. Virol..

[16] Horlen K.P., Schneider P., Anderson J., Nietfeld J.C., Henry S.C., Tokach L.M., Rowland R.R.R. (2007). A cluster of farms experiencing severe porcine circovirus associated disease: Clinical features and association with the PCV2b genotype. J. Swine Health Prod..

[17] Guo L.J., Lu Y.H., Wei Y.W., Huang L.P., Liu C.M. (2010). Porcine circovirus type 2 (PCV2): Genetic variation and newly emerging genotypes in China. Virol. J..

[18] Xiao C.T., Harmon K.M., Halbur P.G., Opriessnig T. (2016). PCV2d-2 is the predominant type of PCV2 DNA in pig samples collected in the U.S. during 2014–2016. Vet. Microbiol..

[19] Nielsen G.B., Nielsen J.P., Haugegaard J., Leth S.C., Larsen L.E., Kristensen C.S., Pedersen K.S., Stege H., Hjulsager C.K., Houe H. (2018). Comparison of serum pools and oral fluid samples for detection of porcine circovirus type 2 by quantitative real-time PCR in finisher pigs. Porc. Health Manag..

[20] Hernandez-Garcia J., Robben N., Magnee D., Eley T., Dennis I., Kayes S.M., Thomson J.R., Tucker A.W. (2017). The use of oral fluids to monitor key pathogens in porcine respiratory disease complex. Porc. Health Manag..

[21] Eddicks M., Koeppen M., Willi S., Fux R., Reese S., Sutter G., Stadler J., Ritzmann M. (2016). Low prevalence of porcine circovirus type 2 infections in farrowing sows and corresponding pre-suckling piglets in southern German pig farms. Vet. Microbiol..

[22] Link E.K., Eddicks M., Nan L., Ritzmann M., Sutter G., Fux R. (2021). Discriminating the eight genotypes of the porcine circovirus type 2 with TaqMan-based real-time PCR. Virol. J..

[23] Segales J., Allan G.M., Domingo M. (2005). Porcine circovirus diseases. Anim. Health Res. Rev..

[24] Dei Giudici S., Mura L., Bonelli P., Hawko S., Angioi P.P., Sechi A.M., Denti S., Sulas A., Burrai G.P., Madrau M.P. (2023). Evidence of Porcine Circovirus Type 2 (PCV2) Genetic Shift from PCV2b to PCV2d Genotype in Sardinia, Italy. Viruses.

[25] Ouyang Y., Nauwynck H.J. (2023). PCV2 Uptake by Porcine Monocytes Is Strain-Dependent and Is Associated with Amino Acid Characteristics on the Capsid Surface. Microbiol. Spectr..

[26] Suh J., Oh T., Park K., Yang S., Cho H., Chae C. (2021). A Comparison of Virulence of Three Porcine Circovirus Type 2 (PCV2) Genotypes (a, b, and d) in Pigs Singularly Inoculated with PCV2 and Dually Inoculated with PCV2 and Porcine Reproductive and Respiratory Syndrome Virus. Pathogens.

[27] Oh T., Cho H., Suh J., Chae C. (2022). Virulence Comparison of Four Porcine Circovirus Type 2 (PCV2) Genotypes (2a, 2b, 2d and 2e) in Pigs Single-Infected with PCV2 and Pigs Dual-Infected with PCV2 and Mycoplasma hyopneumoniae. J. Comp. Pathol..

